# Prognostic Value of Protease Activated Receptor-1 in Children with Acute Lymphoblastic Leukemia

**DOI:** 10.4084/MJHID.2014.029

**Published:** 2014-04-07

**Authors:** Adel A. Hagag, Nahla A. Nosair, Fatma M. Ghaith, Eman H. Elshenawy

**Affiliations:** 1 Department of Pediatrics, Faculty of Medicine. Tanta University. Egypt; 2 Department of Clinical Pathology, Faculty of Medicine. Tanta University. Egypt

## Abstract

**Background:**

Acute Lymphoblastic leukemia (ALL) is a malignant disorder of lymphoid progenitor cells that proliferate and replace the normal hematopoietic cells of the bone marrow. Protease-activated receptors (PARs) comprise a family of trans-membrane G-protein coupled receptors. Protease-activated receptor 1 (PAR-1) is a typical member of this family of receptors that mediate cellular responses to thrombin and related proteases. PAR1 is expressed by a wide range of tumor cells and can promote tumor growth, invasion and metastasis. The aim of this work was to study the role of PAR-1 expression in newly diagnosed ALL patients.

**Patients and methods:**

This study was conducted on 44 children with newly diagnosed ALL who were admitted to Hematology Unit, Pediatric department, Tanta University Hospital including 24 males and 20 females with their age ranged from 4–17 years and their mean age value of 9.06±3.26. All patients were subjected to complete history taking, thorough clinical examination, bone marrow aspiration and flow cytometric analysis for detection of PAR-1 expression by malignant cells.

**Results:**

PAR-1 was positive in 18 cases (41%) and negative in 26 cases (59%) of studied patients. This study showed no significant relation between PAR-1 expression and age, sex and most of the clinical data including hepatomegaly, splenomegaly and purpura while generalized lymphadenopathy was significantly higher in PAR-1 positive group. PAR-1 positive expression was associated with some bad prognostic laboratory parameters including higher hemoglobin, higher white blood cells, higher peripheral blood and bone marrow blast cells, higher serum LDH and lower platelets count. No significant association was detected between PAR-1 expression and immunophenotyping. There were significantly higher remission rates in PAR-1 negative group and significantly higher relapse and death rates in PAR-1 positive group.

**Conclusion:**

From this study, it could be concluded that PAR-1 expression on ALL cells represents an important adverse prognostic factor.

**Recommendations:**

PAR-1 expression should be routinely investigated for better prognostic assessment of ALL patients at diagnosis and should be taken in consideration in designing future therapeutic strategies based on patients- specific risk factors.

## Introduction

Acute lymphoblastic leukemia (ALL) is a malignant disorder of lymphoid progenitor cells that proliferate and replace the normal hematopoietic cells of the bone marrow resulting in a marked decrease in normal blood cell production^1^ and is the most common childhood malignancy, representing nearly one third of all pediatric cancers; the annual incidence is approximately 9–10 cases per 100.000 populations in childhood.^2^ Typically, ALL develops quite quickly (acutely) and rapidly becomes worse unless treated (3) as it spreads into the blood stream and other vital organs quickly.^4^

Many studies over the past 20 years looked at the role of various cellular phenotype assessed at initial diagnosis in predicting therapy response. The associations generally have been strong and are clearly predictive when coupled with several factors such as age, sex, initial hemoglobin level, and total leucocytic and platelets counts.^5^

Protease-activated receptors, (PARs) comprise a family of trans-membrane G- protein coupled receptors that are uniquely activated by proteolytic cleavage of their extracellular portion. This cleavage “unmasks” a new N-terminus, which serves as a “tethered” ligand that binds to the second extracellular domain of the protein, resulting in a variety of cellular responses.^6^ Protease-activated receptor 1 (PAR-1) is a typical member of this family of receptors that mediate cellular responses to thrombin and related proteases.^7^

Physiologically, PAR-1 is expressed by different tissues including vascular cells, neurons, fibroblasts, epithelial cells and others.^8^ PAR-1 has been shown to be overexpressed in various human cancers including breast, melanoma, colon, prostate, ovarian, esophagus and others^9^ and has been associated with several pro-tumoral responses including primary growth, aggressive behavior, invasion, metastasis and angiogenesis.^10^,^11^

PAR-1 is significantly elevated in aggressive leukemias including blast phase of CML and AML subtypes M4/M5, in contrast to chronic phase in CML and CLL. Therefore, this protein plays an important biological role in aggressive leukemias and might offer additional strategies for the development of new therapies.^12^

## Subjects and Methods

This study was done after approval from Ethical Committee of research Center of Tanta University Hospital and written consent from parents of included children in this research and was carried out on 44 children with newly diagnosed ALL who were admitted to Hematology Unit, Pediatric department, Tanta University Hospital including 24 males and 20 females with their age ranged from 4–17 years and their mean age value of 9.06±3.26. ALL was diagnosed according to clinical presentation, morphological, cytochemical smears together with immunophenotyping and was based on the presence of ≥ 20% blast cells in BM according to WHO proposal and MPO negative staining and immunophenotyping results consistent with ALL.^13^ Patients were followed up for 24 months for clinical outcome and fate of the disease.

ALL patients were subjected to the following:

1. Complete history taking
2. Thorough clinical examination: with an especial account on pallor, purpura, hepatomegaly, splenomegaly and lymphadenopathy.
3. Laboratory investigations.

### Specimen collection and handling

Four ml of venous blood were collected using sterile needles through gentle venipuncture after sterilization of the puncture site by alcohol, and collected samples were divided into; one ml was delivered on 20 uL EDTA solution for complete blood count including differential white blood cells count which was done on Leishman stained peripheral blood smear with evaluation using ERMA PCE-210 N cell – counter^14^ and the rest of blood was put in a plain tube and serum was separated for estimation of LDH.

### Bone marrow aspiration

Bone marrow aspiration was performed under complete aseptic technique. Smears of direct bone marrow aspirate were prepared, stained with Lieshman stain for morphologic study and cytochemical stains with Sudan black and Myeloperoxidase and Immunophenotyping using the following panel of fluorescein isothiocyanate/phycoerythrin conjugated monoclonal antibodies:

Lymphoid cell markers.
T-cell markers (CD2, CD3, CD5, CD7).
B-cell markers (CD10, CD19, CD20, CD22).
Myeloid cell markers (CD13, CD33) (15).

### Immunophenotyping for evaluation of PAR-1

One ml of bone marrow or peripheral blood samples (with more than 20% blast cells) were withdrawn on EDTA tubes. Evaluation of PAR-1 was done using Becton Dickinson FAC Scan flow cytometer (BD FACS).^16^ Monoclonal antibodies PAR-1/APC, anti-human reagent for identification of cell expression PAR-1 labeled with fluorescein, commercially available by R&D Systems; FAB3855A. The percentage of blast cells positive for PAR-1 was determined as a percentage from the gated blast cells populations. The negative control was set at 2%. A case was defined as PAR-1 positive if ≥20% of the gated cells expressed PAR-1.^17^

Follow up of patients was done clinically and by blast cell count in the bone marrow (BM) on day 21 after induction chemotherapy which includes: Vincristine 1.5 mg/kg/m^2^/week IV (days 0, 7, 14, 21, 28, 35), Doxorubicin 25 mg/m^2^/week IV infusion (days 0, 7, 14, 21, 28, 35), L-Asparginase 6000 u/m^2^ SC on alternate days for 10 doses, and Prednisone 40 mg/m^2^/day for 6 weeks orally. Bone marrow aspiration was done on day 21. In non-responding cases, we add Etopsoide 100 mg/m^2^/dose IV (days 22, 25, 29), Cyclophosphamide 750 mg/m^2^/dose IV infusion (days 22, 25, 29), Aracytin 100/m^2^/dose IV (days 22, 25, 29), and methotrexate 5g/m^2^ over 4 hours on day 28.^18^

### Definition of complete remission and relapse

Complete remission (CR) is defined as a cellularity of more than 20% with fewer than 5% blasts in bone marrow after induction chemotherapy.^19^ Relapse is defined by the appearance of one of the following: (1) more than 50% lymphoblasts in a single BM aspirate; (2) more than 25% lymphoblasts in BM and 2% or more circulating lymphoblasts; (3) progressive repopulation of lymphoblasts in excess of 5% culminating in more than 25% on two or more BM samples separated by 1 week or more; (4) leukemic cell infiltration in extra medullary organs as gonads; (5) lymphoblasts in CSF with cell count greater than 5 WBCs/mm^3^.^20^

### Statistical analysis

Statistical presentation and analysis of the present study was conducted, using the mean, the standard error, student t- test and Chi- square tests by SPSS V17.

## Results

Table 1 shows no significant differences between PAR-1 positive and PAR-1 negative patients regarding age, sex, pallor, purpura, hepatomegaly and splenomegaly while there was statistically significant difference between PAR-1 positive and PAR-1 negative patients regarding lymphadenopathy with a higher incidence of lymphadenopathy in PAR-1 positive patients.

Table 2 shows statistically significant differences between PAR-1 positive and PAR-1 negative patients regarding hemoglobin and LDH levels, total white blood cells and platelets counts, peripheral blood and BM blast cells percentage with higher hemoglobin and LDH levels, total white blood cells count, peripheral blood and BM blast cells percentage in PAR-1 positive patients while there were significantly lower platelets counts in PAR-1 positive than PAR-1 negative patients and no significant difference between PAR-1 positive and PAR-1 negative patients regarding immunophenotyping.

**Figure 1:**
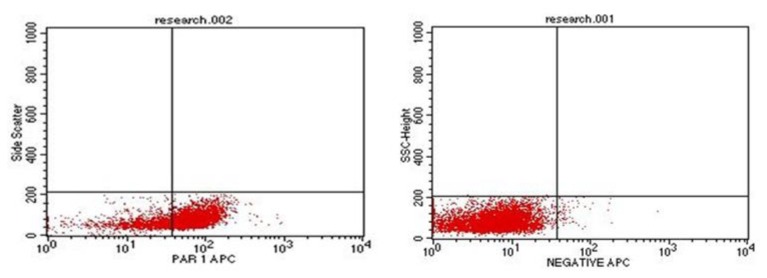
Flow cytometric analysis showing positive case for PAR-1 with high expression (to the left) and negative control for PAR-1 (to the right)

**Figure 2:**
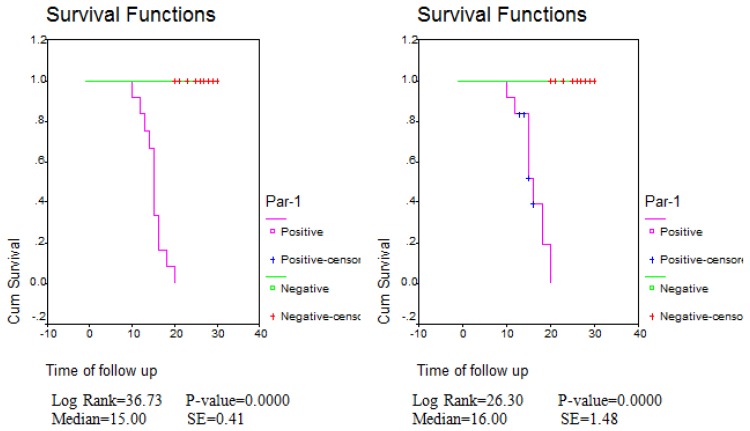
Disease free survival curve (DFS) (to the left) and Overall survival curve (OAS) (to the right). There were statistically significant differences in DFS and OAS between PAR-1 positive and PAR-1 negative groups.

Table 3 shows statistically significant difference between PAR-1 positive and PAR-1 negative expression regarding relapse, death and remission rates with higher relapse and death and lower remission rates in PAR-1 positive group (Fig. 1 and 2).

## Discussion

ALL is the most common childhood malignancy, representing nearly one third of all pediatric cancers. It has become a curable disease in over than 80% of patients with current treatments. However, the treatment of ALL results in significant morbidity and mortality. The use of risk-adapted treatment protocols has improved cure rates while limiting the toxicity of therapy.^21^ PAR-1 plays an important biological role in aggressive leukemias and might offer additional strategies for the development of new therapies.^12^

The present research was done to evaluate the prognostic value of PAR-1 expression in 44 children with newly diagnosed ALL who were admitted to Hematology Unit, Pediatric department, Tanta University Hospital including 24 males and 20 females with their age ranged from 4–17 years and their mean age value of 9.06±3.26 and they included 18 PAR-1 positive patients and 26 PAR-1 negative patients.

There were no significant differences between PAR-1 positive and PAR-1 negative patients regarding age, sex, pallor, purpura, hepatomegaly and splenomegaly while there was statistically significant difference between PAR-1 positive and PAR-1 negative patients regarding lymphadenopathy with a higher incidence of lymphadenopathy in PAR-1 positive patients. These findings were consistent with Mook et al., 2004^22^ who found the same results.

In the present series, there were normocytic normochromic anemia, leukocytosis and thrombocytopenia in studied leukemic patients. This was in agreement with Biswas et al, 2009^23^ who found the same results and explained this by direct result of the diffuse and heavy BM and peripheral blood infiltration due to uncontrolled proliferation of lymphoblasts.

In our study PAR-1 positive expression at diagnosis was significantly associated with bad clinical and laboratory prognostic factors including lymphadenopathy, higher hemoglobin levels, higher white blood cells, higher peripheral blood and bone marrow blast cells and higher serum LDH and lower platelets count. These findings were consistent with Boire et al., 2005^24^ and Salah et al., 2007^11^ who demonstrated that positive PAR-1 expression was associated significantly with various clinicopathologic features and several pro-tumoral responses including primary growth, invasion, lymph node metastasis and depth of tumor invasion and Veiga et al., 2011^12^ who found significantly higher circulating peripheral blood and BM blasts in PAR-1 positive ALL compared to PAR-1 negative cases.

In this study, 81% of patients were B-ALL, and 19% were T-ALL. This was in agreement with Ahmed and Hassab 2008^25^ who found that 83.3% of patients were B-ALL and 14.6% were T-ALL. There were no significant statistical association could be observed between PAR-1 expression and immunophenotyping of ALL. This finding was in agreement with Veiga et al., 2011.^12^

In our study, there were statistically significant differences between PAR-1 positive and PAR-1 negative expression regarding relapse, death and remission rates with higher relapse and death and lower remission rates in PAR-1 positive group. This was in agreement with Veiga et al., 2011 (12) who stated that positive PAR-1 expression was significantly elevated in aggressive leukemias, including blast phase of CML, AML subtypes M4/M5 and B cell ALL in contrast with CML, in chronic phase, and CLL and was associated with poor treatment outcome, Depasquale and Thompson 2008^26^ who demonstrated that PAR-1 expression is a negative prognostic factor in melanomas and strongly correlates with tumor stage and Meis et al 2010^27^ who found decreased long-term survival in PAR-1 expressing patients with lung adenocarcinoma compared with PAR-1 negative patients.

It was found that PAR-1 can promote tumor growth, invasion and metastasis.^24^ In addition PAR-1 activation stimulates proliferation and decreases idarubicin induced cell death in vitro.^28^ The zinc-dependent matrix metalloprotease 1 (MMP-1), also known as interstitial collagenase, has been reported to promote tumor growth and invasion through activation of PAR-1, providing an important link between tumor-generated metalloproteases and PAR-1 expression (Boire et al., 2005).^24^

PAR-1 plays a primary role in the process of metastasis by stimulating the secretion of matrix metalloproteinase by virtue of their ability to degrade the extracellular matrix (ECM) barrier. However, MMPs are also capable of cleaving non-ECM molecules. The protease-activated receptors (PARs) are the latest MMP targets. The thrombin receptor PAR-1 has now been shown to be cleaved and activated on the tumor cell surface by stromal-derived MMP1. The resulting PAR1 activates intracellular G proteins to turn on the migratory and invasive program in tumor cells. This MMP-PAR axis may represent a novel signaling pathway communicating between tumor and stromal cells during tumor progression.^29^

## Conclusion

PAR-1 expression on ALL cells represents an important adverse prognostic factor and therefore its expression should be routinely investigated for better prognostic assessment of ALL patients at diagnosis and should be taken in consideration in designing future therapeutic strategies based on patient- specific risk factors.

## Figures and Tables

**Table 1:** Clinical characteristics of studied ALL patients.

	PAR-1 positive (No= 18)	PAR-1 negative (No= 26 )	T value P value	

Age				
Range	5–17	4–15	0.245	0.24
Mean ± SD	8.75±3.56	9.17±3.28		

Sex				
Males	10	14	0.023	0.880
Females	8	12		

Pallor	12	18	0.052	0.819

Purpura	14	21	3.772	0.052

Hepatomegaly	16	23	1.250	0.263

Splenomegaly	15	22	0.052	0.819

Lymphadenopathy	16	12	11.699	0.001*

* Highly significant

**Table 2:** Laboratory data of studied ALL patients.

	PAR-1 positive (No = 18)	PAR-1 negative (No=26 )	T value P value	

Hemoglobin (gm/dl)				
Range	8.2–11.7	5.8–9.2	2.83	0.01*
Mean ± SD	9.32±1.67	6.13±1.06		

WBCs (×10^3^/mm^3^)				
Range	25–180	11–150	4.74	<0.01*
Mean ± SD	124.90±48.21	47.43±40.78		

Platelets (×10^3^/mm^3^)				
Range	10–70	13–80	2.06	0.04*
Mean ± SD	34.83±22.21	53.21±25.06		

PB blasts (%)				
Range	39–80	8–60	6.09	<0.01*
Mean ± SD	61.71±13.10	25.03±17.84		

BM blasts (%)				
Range	38–94	25–87	4.22	<0.01*
Mean ± SD	74.08±16.24	43.57±21.18		

LDH (U/L)				
Range	570–2100	460–620	2.87	0.02*
Mean ± SD	937.58±493.6	527.64±52.2		

Immuno-phenotyping				
B- cell ALL	15	21	0.978	0.322
T- cell ALL	3	5		

* Significant

**Table 3:** Prognostic value of PAR-1 positive expression in ALL patients.

PAR-1 expression		Patients outcome ( No=44)		
		Death	Remission	Relapse
PAR-1 Negative (No=26) (59%)		1 (2.2)	21 (47.7)	4 (9)
PAR-1 Positive (No=18) (41%)		7 (15.9)	6 (13.6)	5 (11.3)
Total Number = 44 (100%)		8 (18.18)	27 (61.3)	9 (20.45)
Chi-square	X^2^	28.015		
	p-value	<0.001*		

* Highly significant (p<0.01)
